# Biomarkers of persistent renal vulnerability after acute kidney injury recovery

**DOI:** 10.1038/s41598-021-00710-y

**Published:** 2021-10-27

**Authors:** Isabel Fuentes-Calvo, Cristina Cuesta, Sandra M. Sancho-Martínez, Omar A. Hidalgo-Thomas, María Paniagua-Sancho, Francisco J. López-Hernández, Carlos Martínez-Salgado

**Affiliations:** 1 Institute of Biomedical Research of Salamanca, IBSAL, Edificio Departamental, S-18, Campus Miguel de Unamuno, 37007 Salamanca, Spain; 2 Translational Research on Renal and Cardiovascular Diseases (TRECARD)-REDINREN (ISCIII), Department of Physiology and Pharmacology, University of Salamanca, Salamanca, Spain

**Keywords:** Kidney, Physiology, Biomarkers, Nephrology

## Abstract

Acute kidney injury (AKI) is a risk factor for new AKI episodes, chronic kidney disease, cardiovascular events and death, as renal repair may be deficient and maladaptive, and activate proinflammatory and profibrotic signals. AKI and AKI recovery definitions are based on changes in plasma creatinine, a parameter mostly associated to glomerular filtration, but largely uncoupled from renal tissue damage. The evolution of structural and functional repair has been incompletely described. We thus aimed at identifying subclinical sequelae persisting after recovery from cisplatin-induced AKI in rats. Compared to controls, after plasma creatinine recovery, post-AKI kidneys showed histological alterations and attendant susceptibility to new AKI episodes. Tubular function (assessed by the furosemide stress test, FST) also remained affected. Lingering parenchymal and functional subclinical alterations were paralleled by tapering, but abnormally high levels of urinary albumin, transferrin, insulin-like growth factor-binding protein 7 (IGFBP7), tissue inhibitor of metalloproteinases-2 (TIMP-2) and, especially, the [TIMP-2]*[IGFBP7] product. As subclinical surrogates of incomplete renal recovery, the FST and the urinary [TIMP-2]*[IGFBP7] product provide two potential diagnostic tools to monitor the sequelae and kidney vulnerability after the apparent recovery from AKI.

## Introduction

The acute kidney injury (AKI) syndrome is characterized by an abrupt decline in renal function during a period of seven days or less, which is associated with high morbidity and mortality rates^1,2^. The incidence of AKI has increased in the last decade along with factors such as population aging and associated diseases (e.g. diabetes and hypertension), and also due to the use of stricter definitions of AKI. It is estimated that AKI occurs in 20–200 people per million inhabitants and affects 7–18% of hospitalized patients, a percentage that increases up to 50% in patients admitted to intensive care units^2–4^. AKI has traditionally been considered a mostly reversible event with no consequences for long-term renal function. However, epidemiological studies have recently shown that AKI is a risk factor for the eventual loss of kidney function, for the progression to chronic kidney disease (CKD), and for other adverse outcomes such as cardiovascular events and death^2,5–7^.

AKI may be preferentially caused by hemodynamic deregulation with no involvement of renal structures (i.e. pre-renal AKI), or by damage to renal tissues (i.e. intrinsic AKI). A most common form of intrinsic AKI is acute tubular injury (formerly known as acute tubular necrosis), an affection of the tubular compartment often involving epithelial cell death. Although kidneys usually undergo complete functional recovery after an AKI episode due to the effective regenerative capacity of tubular cells, under undetermined circumstances renal repair becomes maladaptive leading to proinflammatory and profibrotic signals, which eventually evolve to CKD^8,9^. Accordingly, monitoring the repair process by means of non-invasive technology is an unmet but necessary need to better handle AKI with precision medicine criteria.

An ideal definition of repair should be based on both restoration of kidney structure and recovery of renal function. A consensus definition of AKI recovery exists, which is based on the normalization (or return to basal levels) of plasma creatinine (pCr)^3,10^. However, this definition carries the inherent limitations of pCr. pCr increases over the normality range only when the function corresponding to at least 50–70% of nephrons has been lost. Consequently, after an AKI episode pCr may return to basal levels in the presence of significant structural alterations^3,11–13^. The limitations of pCr to assess renal recovery are reinforced by epidemiological data showing an association between AKI and progression to CKD even when pCr has returned to basal levels^14,15^. Thus, it is critical to develop complementary methods to detect subclinical (i.e. under normal or basal pCr) renal alterations after an AKI episode.

During the last two decades, new biomarkers have been identified that detect kidney damage independently of pCr, even in subclinical circumstances. A few studies have explored the use of these biomarkers [i.e. insulin-like growth factor binding protein 7 (IGFBP-7), kidney injury molecule-1 (KIM-1), neutrophil gelatinase-associated lipocalin (NGAL), tissue metalloproteinase 2 (TIMP-2), interleukin 18, liver-type fatty acid-binding protein, *N*-acetyl-β-*d*-glucosaminidase], to monitor the recovery process^9,12^. Still, structural and functional recovery in the days and weeks immediately following the AKI episode has not yet been sufficiently characterized. Post-AKI monitoring is necessary because AKI can evolve to Acute Kidney Disease (AKD), a condition in which AKI stage 1 or higher remains ≥ 7 days after an AKI event. Of special interest is the stage 0A of AKD in which pCr has returned to basal levels and there is no evidence of injury or functional loss, but patients still retain a risk of further kidney damage^2^. AKD provides an opportunity window to prevent future renal adverse outcomes^2,16^ and, therefore, new diagnostic technology is needed to identify patients in early AKD.

The objective of this study was to characterize the evolution of renal repair and potential functional and structural sequelae after pCr had returned to baseline levels following an episode of AKI in rats. In particular, we aimed at evaluating whether and for how long animals retain enhanced sensibility to AKI (i.e. higher risk of AKI) after pCr recovery and, if so, whether such a condition may be detected by known biomarkers of subclinical renal damage.

## Results

### The kidneys show persisting structural alterations after functional recovery from AKI

**Figure 1:**
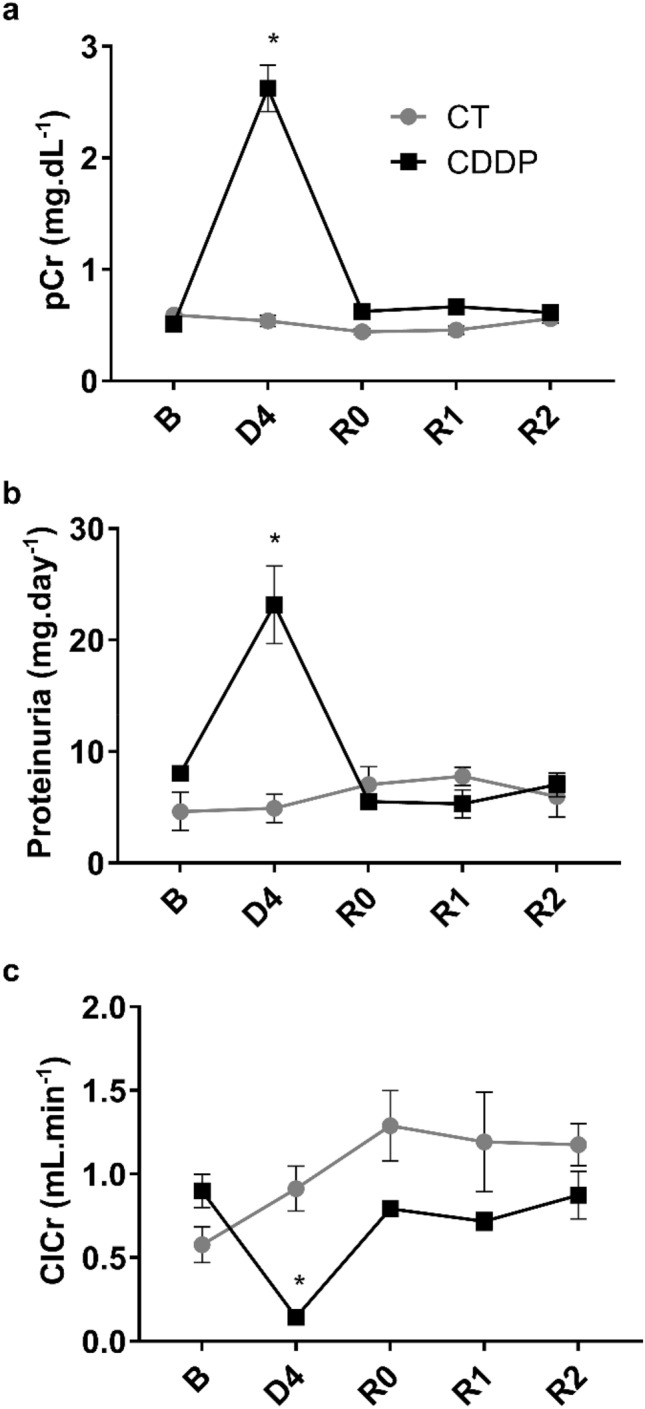
Renal function parameters: plasma creatinine (pCr) (**a**), proteinuria (**b**) and creatinine clearance (ClCr) (**c**). *B* basal, *CDDP* cisplatin treatment (5 mg kg^−1^ body weight) group, *CT* control group, *D4* day of maximum kidney damage after cisplatin treatment, *R0* day of recovery, *R1* 1 week after recovery; *R2* 2 weeks after recovery. *P* < 0.01: * versus B.

**Figure 2:**
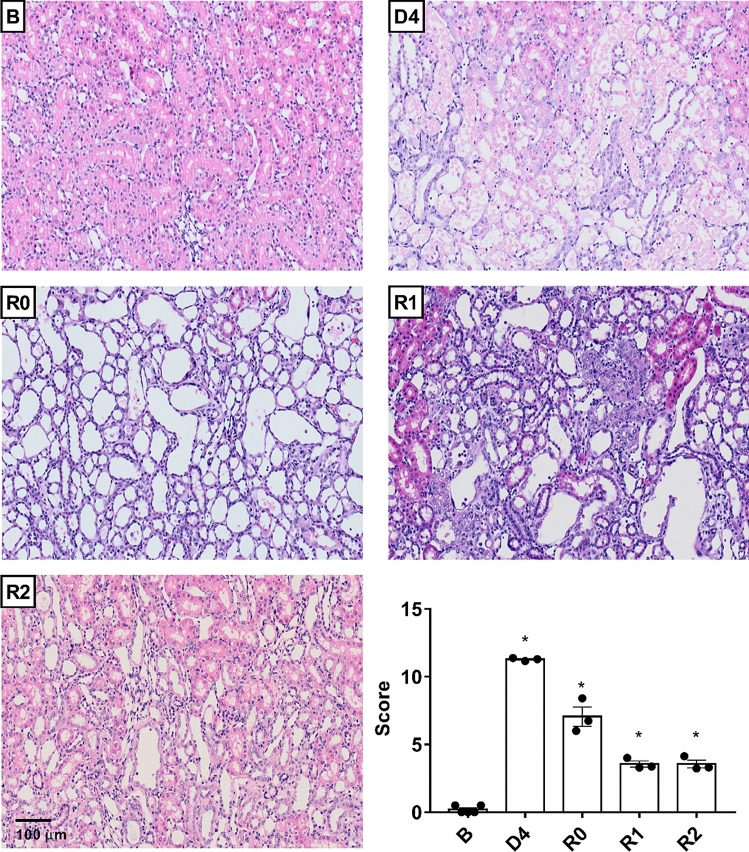
Renal histology: histological damage. Hematoxylin–eosin staining in control group, D4, R0, R1 and R2; quantification of histological damage (score). *P* < 0.01: * versus B.

**Figure 3:**
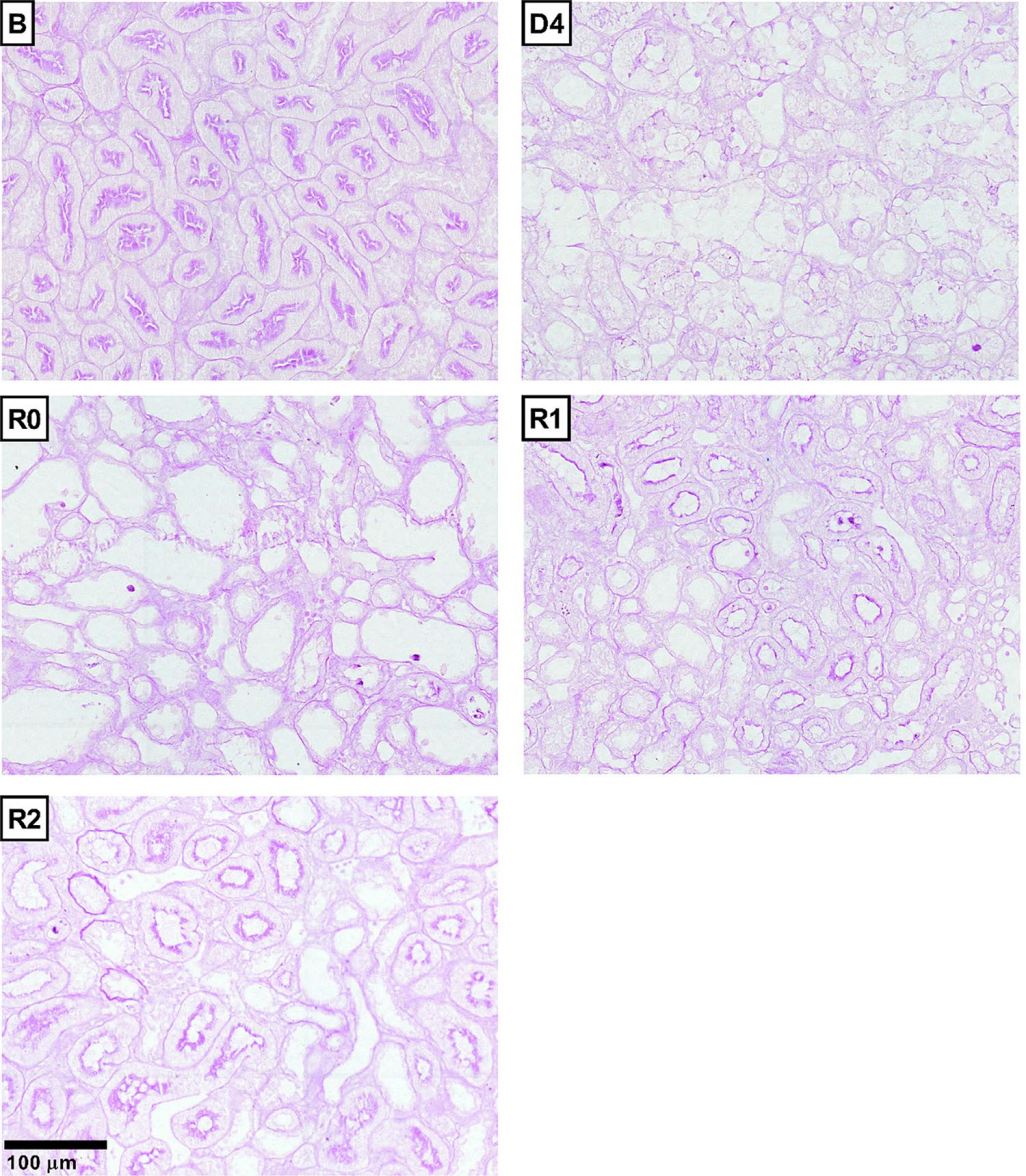
Renal histology: brush border membrane disorganization. Periodic Acid-Schiff staining in control group, D4, R0, R1 and R2.

Cisplatin treatment (5 mg kg^−1^, i.p.) induces significant increases in plasma creatinine levels (Fig. 1a), proteinuria (Fig. 1b) and a decrease in creatinine clearance (Fig. 1c) after 4 days (D4); these parameters return to baseline values about 4–6 days after D4 (R0). By D4, cisplatin treatment induces severe structural alterations in the kidneys that include cast formation, tubular necrosis and tubular dilatation, as observed by haematoxylin–eosin staining (Fig. 2). In kidneys from rats with normalized renal function under traditional criteria (i.e. at R0, R1 and R2), when plasma creatinine, proteinuria and creatinine clearance have returned to basal levels, structural alterations, mainly tubular dilatation, are still present, although to a lower and tapering degree (Fig. 2). After quantification of tissue damage, we noticed that these structural alterations remain up to 2 weeks (i.e. R2) after apparent recovery of renal function (Fig. 2). Periodic acid-Schiff staining shows that the tubular epithelium brush border membrane is completely disorganized or absent in D4, and that situation does not improve significantly when renal function parameters are normalized by R0 (Fig. 3). The brush border membrane shows mild regeneration at R1 and R2, although significant damage remains, as compared with the normal structure observed in basal conditions or in control animals (Fig. 3). Summarizing, the histological analysis shows that there are remarkable structural alterations in the renal tissue after an episode of cisplatin-induced AKI, which persist when the renal function parameters return to baseline values.

### Rats retain increased susceptibility to a new AKI after plasma creatinine normalization

**Figure 4:**
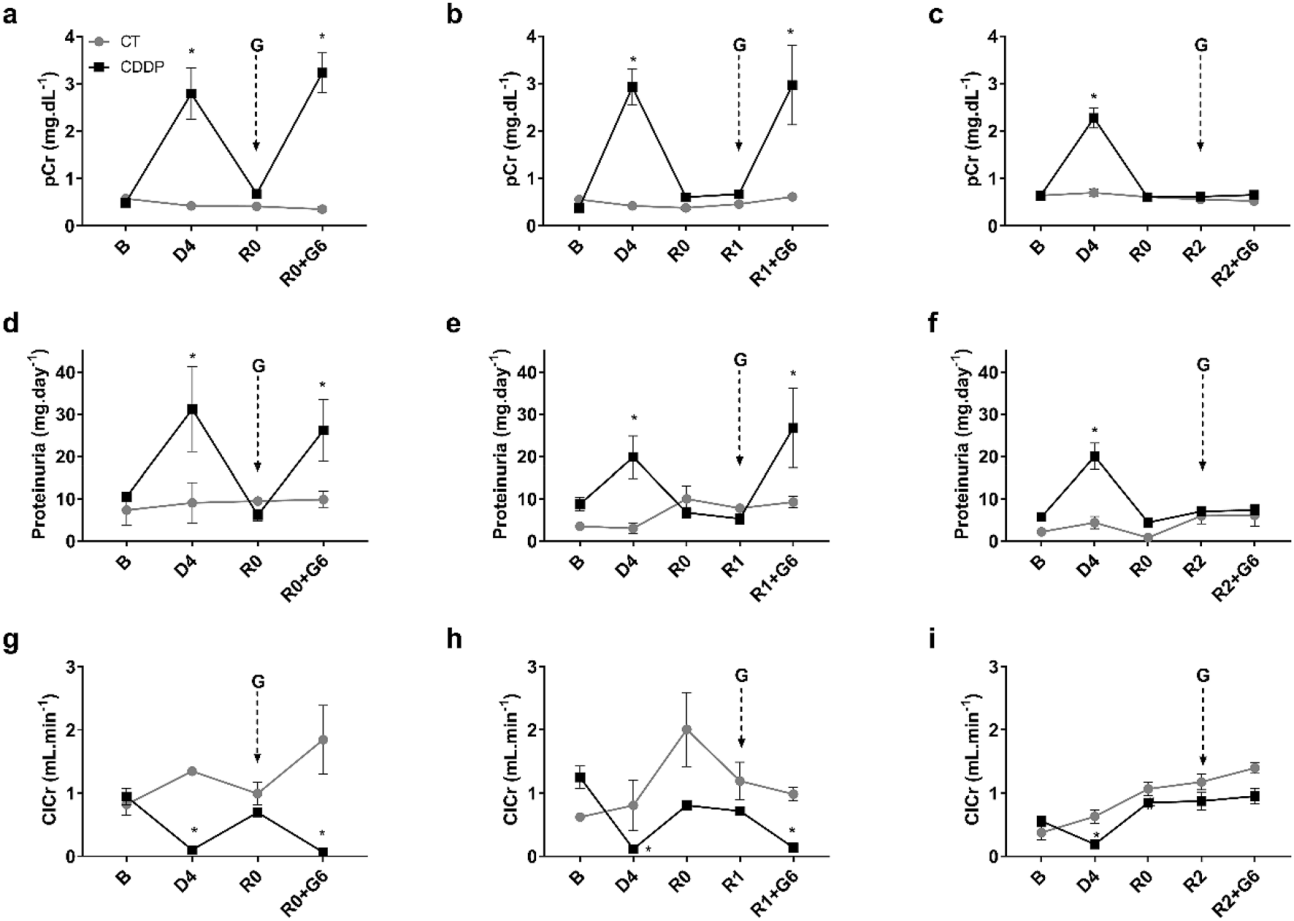
Susceptibility to AKI: plasma creatinine (pCr) (**a**–**c**), proteinuria (**d**–**f**) and creatinine clearance (ClCr) (**g**–**i**). *B* basal, *CDDP* cisplatin treatment (5 mg kg^−1^ body weight) group, *CT* control group, *D4* day of maximum kidney damage after cisplatin treatment, *G* gentamicin administration, *R0* day of recovery, *R0* + *G6* 6 days of gentamicin treatment (50 mg kg^−1^ body weight) after R0, *R1* 1 week after recovery, *R1* + *G6* 6 days of gentamicin treatment (50 mg kg^−1^ body weight) after R1, *R2* 2 weeks after recovery, *R2* + *G6* 6 days of gentamicin treatment (50 mg kg^−1^ body weight) after R2. *P* < 0.01: * versus B.

In rats that have undergone a previous cisplatin-induced AKI, treatment with the aminoglycoside gentamicin in subtoxic doses (50 mg kg^−1^.day^−1^ for 6 days, i.p.) promotes significant new increases in plasma creatinine levels and proteinuria and a decrease in creatinine clearance when gentamicin is administered the day of recovery (i.e. at R0) or one week after the day of recovery (i.e. at R1). However, this increase is not observed when subtoxic treatment is started 2 weeks after the recovery of cisplatin-induced AKI (i.e. at R2), nor in animals that have not had a previous AKI (CT group) (Fig. 4). These data suggest that after a cisplatin-induced AKI episode, the kidneys remain in a state of increased susceptibility or predisposition to suffer a new AKI. Although renal susceptibility is lost 2 weeks after recovery from AKI, renal tissue structure is still under repair. In fact, tapering but significant alterations are still evident.

### Alterations in the furosemide stress test parallel post-AKI predisposition to AKI

**Figure 5:**
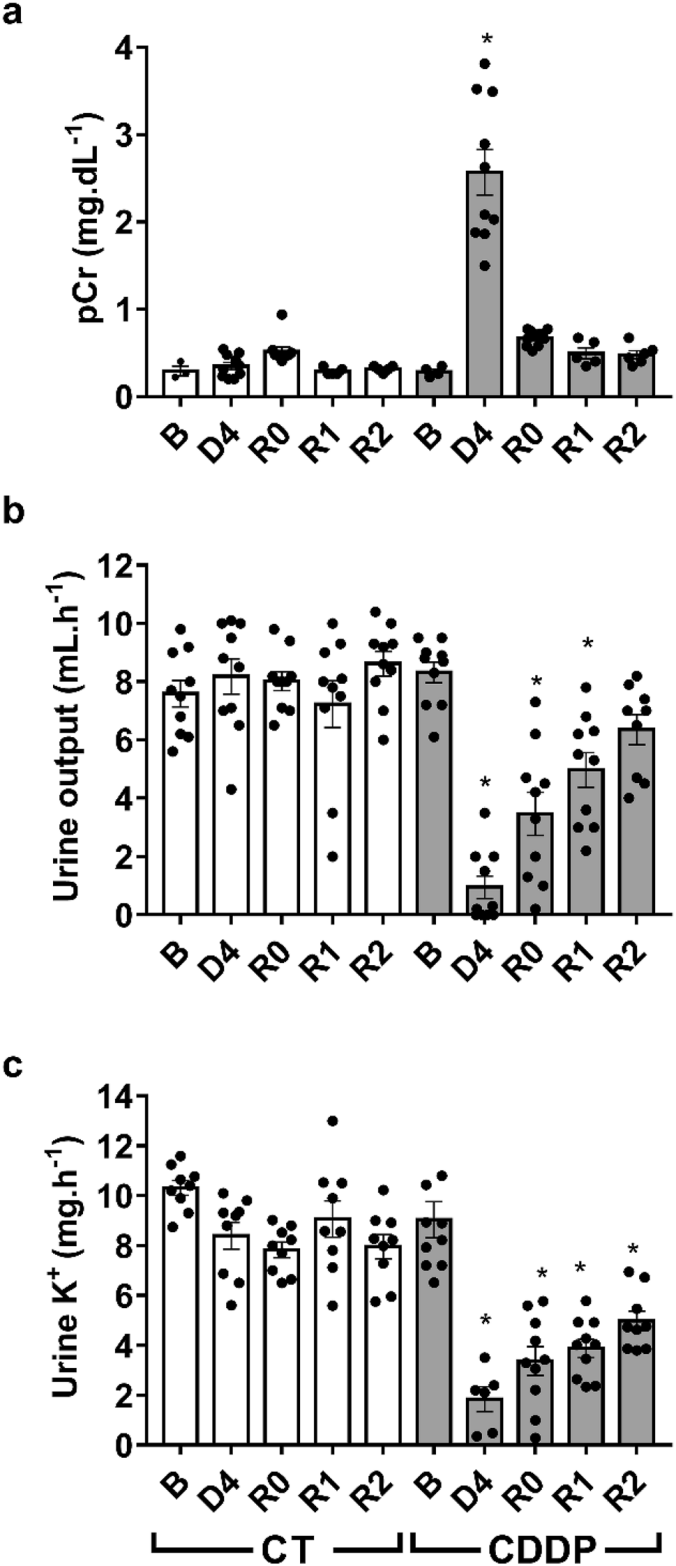
Furosemide stress test. Plasma creatinine (pCr) (**a**), urine output (**b**) and urinary potassium excretion (**c**) after administration of furosemide (20 mg kg^−1^, i.p.). *B* basal, *CDDP* cisplatin treatment (5 mg kg^−1^) group, *CT* control group, *D4* day of maximum kidney damage after cisplatin treatment, *R0* day of recovery, *R1* 1 week after recovery, *R2* 2 weeks after recovery. *P* < 0.01: * versus B.

For its sensitivity to detect minimal and subclinical tubular alterations^17^, we applied the FST during the AKI episode and after pCr normalisation. Figure 5 shows that a profoundly reduced response to the FST (decreased urine output and urinary K^+^ excretion) is produced by cisplatin during AKI. This alteration progressively tapers off after AKI, but FST remains significantly reduced after pCr normalisation and up to two weeks thereafter, coinciding with the timing of the predisposition to AKI.

### Urinary biomarkers detect subclinical damage after functional recovery from AKI, and correlates with new AKI episodes before subtoxic treatment with gentamicin

**Figure 6:**
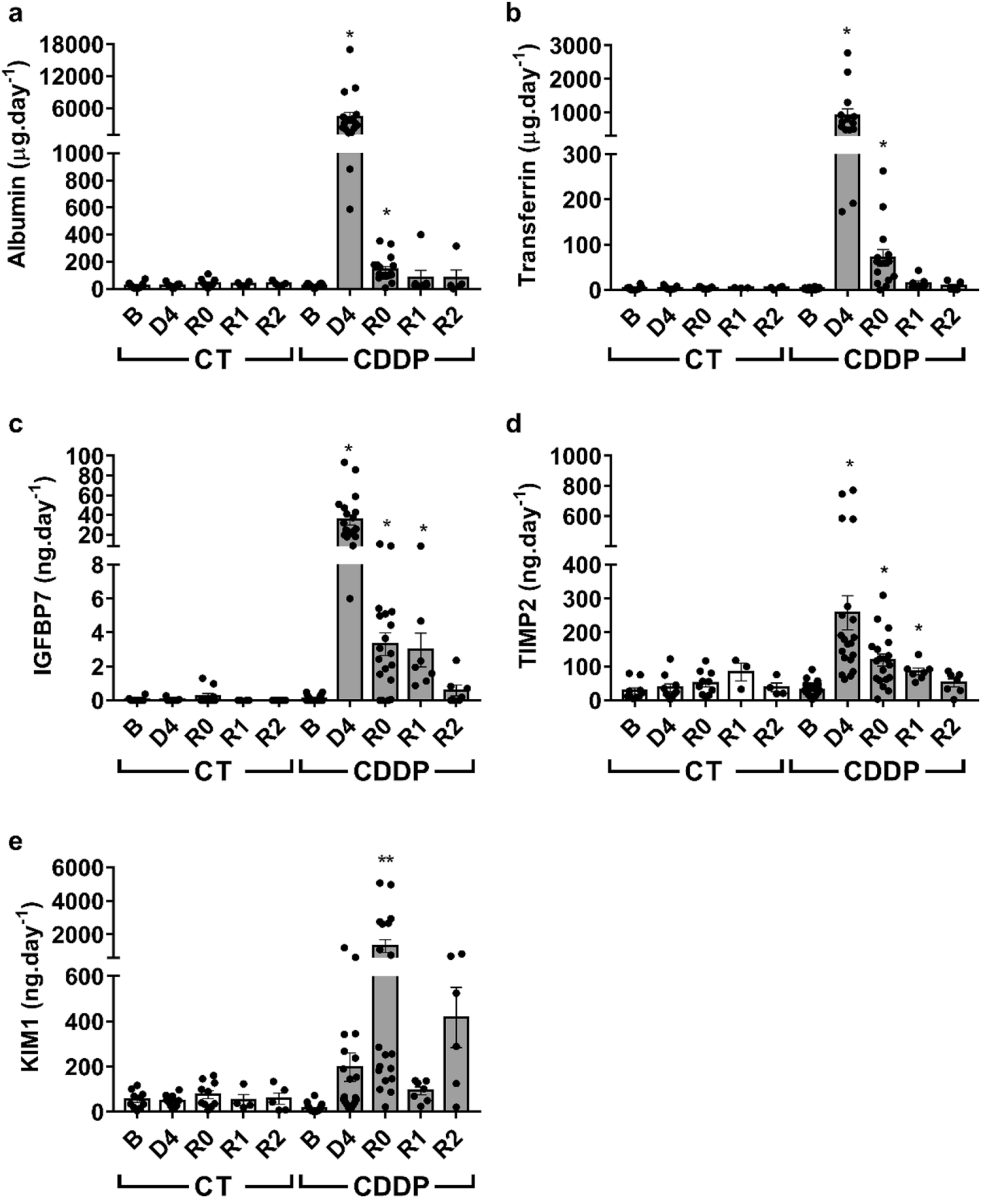
Urinary excretion of biomarkers. Albumin (**a**), transferrin (**b**), IGFBP7 (**c**), TIMP-2 (**d**) and KIM-1 (**e**) urinary excretion. *B* basal, *CDDP* cisplatin treatment (5 mg kg^−1^ body weight) group, *CT* control group, *D4* day of maximum kidney damage after cisplatin treatment, *R0* day of recovery, *R1* 1 week after recovery, *R2* 2 weeks after recovery. *P* < 0.05: * versus B; *P* < 0.01: ** versus B.

Cisplatin treatment promotes the urinary excretion of significant levels of albumin, transferrin, IGFBP7, TIMP-2 and KIM-1. The highest urinary excretion of these biomarkers is mostly detected in D4, except for KIM-1 that peaks at R0. Although progressively declining, all these markers are still present at significant levels in urine when plasma creatinine is normalized (R0), and even two weeks later (Fig. 6).

**Figure 7:**
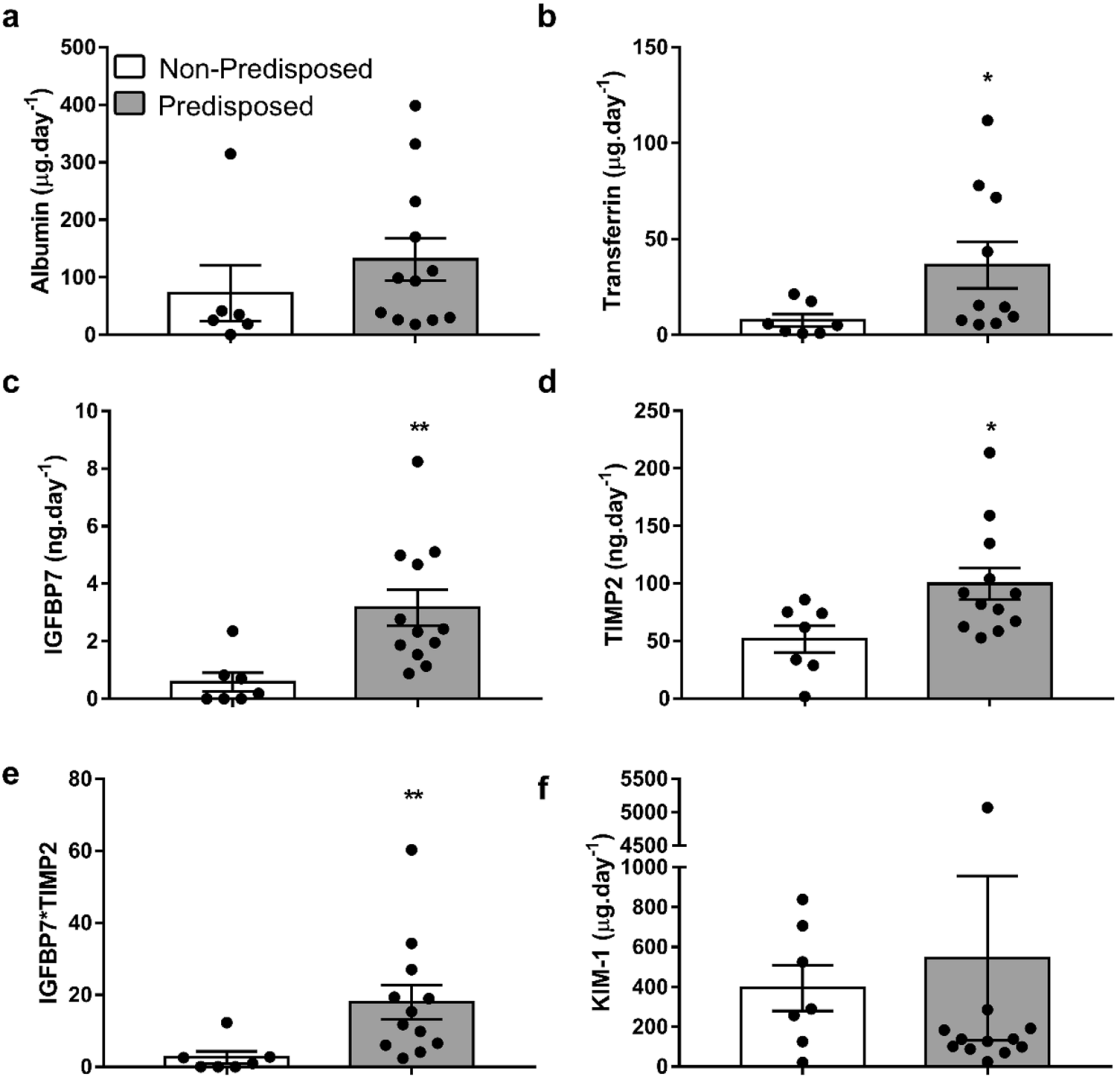
Increased levels of urinary biomarkers in rats predisposed to new AKI episodes. Albumin (**a**), transferrin (**b**), IGFBP7 (**c**), TIMP-2 (**d**), IGFBP7*TIMP-2 (**e**) and KIM-1 (**f**) urinary excretion in rats with and without predisposition to a new AKI episode. *P* < 0.01: ** or *P* < 0.05: * versus non-predisposed.

**Figure 8:**
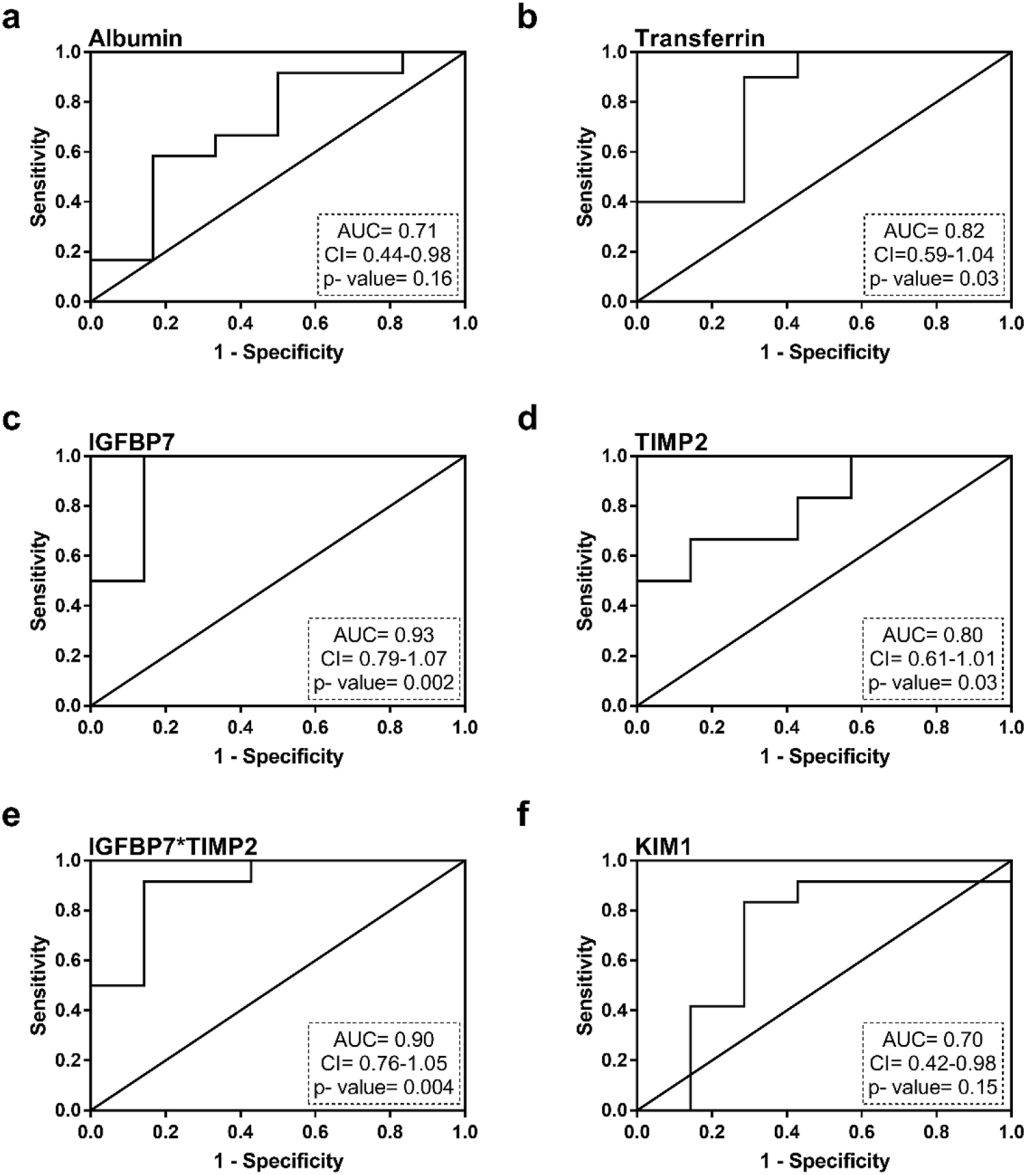
ROC curves showing predictive value of urinary biomarkers on increased susceptibility to new AKI. *AUC* area under the curve, *CI* confidence interval.

After recovering from a first AKI episode, the urinary excretion of transferrin, IGFBP7, TIMP-2 and IGFBP7*TIMP-2 is significantly higher in rats predisposed to suffer new AKI episodes (R0, R1) than in those who do not present this predisposition (R2) (Fig. 7b–e), while there are no changes in urinary albumin excretion in rats predisposed to a new AKI (Fig. 7a). The receiver operating characteristic (ROC) curves in Fig. 8 show the predictive capacity of each biomarker to identify rats predisposed to suffer a new AKI after subtoxic treatment with gentamicin, independently of the moment considered after AKI recovery. Interestingly, IGFBP7, IGFBP7*TIMP-2, transferrin and TIMP-2 show areas under the curve (AUC) of 0.93, 0.90, 0.82 and 0.80, respectively (Fig. 8). Furthermore, there are highly significant correlations between the urinary levels of these markers at the predisposition stage and the magnitude of the subsequent decline in renal function produced after treatment with subtoxic doses of gentamicin (i.e. increment in pCr and reduction in ClCr seen at R0 + G6 and R1 + G6) (Table 1).

**Table 1:** Correlation of urinary biomarker levels at the predisposition stage with the severity of the subsequent AKI.

	Biomarker R versus PCr R + G6		Biomarker R versus ClCr R + G6	
	r-Spearman	*p*-valor	r-Spearman	*p*-valor
Albumin	0.1611	0.515	− 0.1456	0.564
Transferrin	0.6324	**0.008**	− 0.5172	**0.035**
IGFBP7	0.6655	**0.002**	− 0.6989	**0.001**
TIMP2	0.6684	**0.002**	− 0.6117	**0.005**
IGFBP7*TIMP2	0.6397	**0.003**	− 0.7068	**0.001**
KIM1	− 0.0912	0.701	0.1079	0.660

Spearman’s correlations between urinary levels of biomarkers in recuperation points (R0, R1, R2) with the increase in plasma creatinine and the decrease in creatinine clearance determined after 6 days of subtoxic gentamicin treatment (50 mg kg^−1^ body weight) (R0 + G6, R1 + G6, R2 + G6) started at every recuperation point.

In bold, statistically significant differences.

## Discussion

Our study shows that after the apparent clinical recovery from an AKI episode (that is, when pCr and ClCr have returned to baseline levels), the kidney’s structure remains affected, at least for two weeks in the rat. These lingering structural alterations coincide with an increased subclinical predisposition to new renal damage, and with an altered FST response and an increased urinary excretion of tubular injury biomarkers, including transferrin, IGFBP7 and TIMP-2.

The pathophysiological profiling and diagnosis of the condition known as predisposition to suffer AKI is a research field of clinical relevance. Several scoring models have been developed in the last 15 years, which predict the need of dialysis following surgical procedures with areas under the receiver operating characteristic (ROC) curve of about 0.8 based on demographic and clinical variables^18,19^. However, additional tools are needed to predict milder AKI not requiring dialysis, which is frequently involved in a variety of in-hospital outcomes. It was previously identified a pCr-negative, AKI-predisposing condition induced by sub nephrotoxic regimes of several drugs and toxins, including cisplatin^17,20–22^. In fact, these drug regimens cause absolutely no evidence of renal injury per se. However, there are no studies assessing the functional risk or predisposition to suffer a new AKI bout after the apparent recovery from a previous episode. Our results indicate that, whilst basal renal function is apparently normal, increased subclinical, post-AKI susceptibility to new AKI remains beyond pCr normalization, coinciding with incomplete structural recovery and with the presence of injury biomarkers in the urine. Monitoring repair by means of a non-invasive liquid biopsy may have high clinical repercussion, because repeated AKI is now known to be tightly associated with progression to AKD and CKD^2,23–25^. This is stressed by the fact that the gold standard biomarker of AKI (i.e. pCr) is not suitable to monitor repair, as its normalization does not preclude structural and functional sequelae^26^. It is thus of great clinical interest to pre-emptively identify individuals at increased risk of further AKI (even of subclinical AKI), for optimal, preventive and bespoke clinical handling. Accordingly, there is thus an urgent need of identifying non-invasive biomarkers of predisposition to new kidney damage.

Interestingly, we found several subclinical markers that correlate not only with structural sequelae, but also with the extant predisposition to AKI associated with them. A first marker is the response to the FST, which is altered in predisposed rats after pCr normalization. The loop diuretic furosemide inhibits the luminal Na–K–Cl cotransporter in the thick ascending limb of the loop of Henle and binds to the chloride transport channel, and causes sodium, chloride, and potassium loss in urine, and osmotic diuresis. Previously, our research group has shown that the FST identifies subclinical renal alterations associated with predisposition to AKI in the proximal tubule and thick ascending limb. In that study, predisposed animals by a sub-nephrotoxic dose of cisplatin (i.e. no damage takes place) showed reduced furosemide-induced diuresis and K^+^ excretion^17^. In the present study, we observed that, during two weeks after overt damage has occurred, the urine output and K^+^ urinary excretion forced by furosemide are also significantly reduced, thus denoting that there are still alterations in tubular function that mostly affect its response capacity (i.e. the tubular functional reserve). In agreement with this, as it was previously shown^17^, the FST is a very sensitive tubule injury test. Accordingly, the FST potentially provides a very useful tool for detecting subtle alterations, but additional biomarkers are needed to inform damage granularity. In this sense, along with an altered FST, we identified increased urinary excretion of albumin, transferrin, IGFBP7 and TIMP-2 in connection with AKI sequelae and, some of them with the resulting predisposition to new AKI episodes.

These biomarkers are useful for the early and subclinical (i.e. with normal pCr) detection of AKI, and for the estimation of AKI prognosis and evolution. Albuminuria is a known AKI biomarker^27^, independently associated with a lower rate of AKI recovery in critically ill septic patients^28^. Increased albuminuria may be the consequence of glomerular or tubular alterations^29,30^. In our model, because of cisplatin’s mechanisms of nephrotoxicity^17,31^, albuminuria probably results from altered proximal tubular reclamation. The meaning of urinary transferrin reinforces this concept. In fact, our group has recently shown that urinary transferrin is a marker of tubular damage, and associates to the predisposition to renal damage induced by nephrotoxic insults causing subclinical tubular alterations, and not to other types of damage (i.e. vascular, haemodynamic)^32^. The combination of [TIMP-2]*[IGFBP7], urinary markers of G1 cell cycle arrest, improve the identification of patients at risk for imminent AKI^33,34^, and the decline in their urinary values is a valid predictor for renal recovery^35^. The exact mechanism by which IGFBP7 and TIMP-2 levels increase in the urine in different AKI models is not completely known, although increased filtration, proximal tubular cell leakage and defects in tubular reabsorption are the most likely mechanisms^36^.

Our results also evidence the distinct biological role, and thus the potential diagnostic meaning, of each of the so-called renal injury-biomarkers. Renal injury is an ambiguous term encompassing a variety of alterations, each of which yielding specific markers. A challenge is thus to associate individual biomarkers to concrete injury patterns, mechanisms or events, for a more specific, pathophysiological and personalized diagnosis of AKI. For instance, in our model the altered FST and the increased urinary excretion of transferrin, TIMP-1 and IGFBP7 all seem to portray a common pathological meaning, and thus indirectly link the observed post-AKI sequelae with the resulting predisposition to AKI. Conversely, urinary albumin and KIM-1 show some association with sequelae but not with predisposition, indicating that the undetermined (and probably different) pathological events producing their appearance in the urine in this model are not linked to the increased sensitivity to new AKI.

In conclusion, this study shows that when plasma creatinine and creatinine clearance return to baseline values after an intrinsic AKI episode, both kidney structure and function may not be fully recovered. Our results suggest that the follow-up of urinary transferrin, albumin and [TIMP-2]*[IGFBP7]), along with the FST, might be useful for monitoring AKI sequelae and incomplete AKI repair. On the other hand, specifically transferrin, IGFBP7, TIMP-2, [TIMP-2]*[IGFBP7] and the FST also detect the increased predisposition to suffering a new episode of AKI, which may be used to pre-emptively identify patients at higher risk for appropriate and personalized handling.

## Methods

All reagents were purchased from Merck (Madrid, Spain), except where otherwise indicated.

### In vivo experimental model

Animals were treated in accordance with ARRIVE guidelines and the Principles of the Declaration of Helsinki and the European Guide for the Care and Use of Laboratory Animals (Directive 2010/63/UE) and Spanish national and regional regulations (Law 32/2007/Spain, RD 1201/2005 and RD 53/2013). The Bioethics Committee of the University of Salamanca approved all procedures for Animal Care and Use. Male Wistar rats (240–260 g) were maintained under controlled environmental conditions, fed on standard chow and allowed to drink water ad libitum.

#### Animal model 1: evaluation of subclinical damage after AKI

Rats were subdivided into two groups (n = 6 per group and time point): control group (CT): rats receiving saline solution (0.9% NaCl, i.p.); cisplatin group (CDDP): rats receiving cisplatin (5 mg kg^−1^, i.p.), as in our previous studies^37^. Renal function was evaluated in the following days: Basal (B), immediately prior to cisplatin administration; day of maximum kidney damage (D4; i.e. the day of highest pCr^17^); day of recovery (R0; i.e. the first day in which pCr returns to basal levels); and one and two weeks after R0 (R1 and R2 respectively) (Supplementary Figure 1).

#### Animal model 2: evaluation of predisposition to new AKI episodes

This experimental model was performed to assess if rats were predisposed to suffer new AKI episodes when pCr had already returned to basal levels after a previous AKI episode. We used our previously published model, in which predisposition to AKI is unmasked by treating rats with a sub-nephrotoxic dosage regime of gentamicin^20,21,32^. In this model, only predisposed rats (and not controls) undergo AKI when subject to sub-nephrotoxic gentamicin.

Rats were subdivided into 6 groups (n = 6 each): cisplatin + gentamicin R0 group (R0 + G6): rats receiving cisplatin (5 mg kg^−1^, i.p.) + gentamicin (50 mg kg^−1^ day^−1^ for 6 days, i.p., starting the first day of recovery, R0); cisplatin + gentamicin R1 group (R1 + G6): rats receiving cisplatin (5 mg kg^−1^, i.p.) + gentamicin (50 mg kg^−1^ day^−1^ for 6 days, i.p., starting 1 week after recovery, R1); cisplatin + gentamicin R2 group (R2 + G6): rats receiving cisplatin (5 mg kg^−1^, i.p.) + gentamicin (50 mg kg^−1^ day^−1^ for 6 days, i.p., starting two weeks after recovery, R2); 3 gentamicin control groups (CT G6): rats receiving saline solution (i.p.) + gentamicin (50 mg kg^−1^ day^−1^ for 6 days, i.p., starting in R0, R1 and R2) (Supplementary Figure 1).

### Sample collection

At selected time points (Supplementary Figure 1), urine and plasma samples were collected to evaluate renal function. For urine collection, rats were individually allocated in metabolic cages. 24-h urine was collected, centrifuged and stored at -80 °C. Blood was drawn from the tail vein, centrifuged and plasma was stored at -80 °C. Immediately before sacrifice (50 mg kg^−1^ sodium pentobarbital i.p.), kidneys were perfused by the aorta with saline solution and dissected. One-half was frozen in liquid nitrogen and subsequently kept at − 80 °C. The other half was fixed in buffered 3.7% p-formaldehyde for histological studies.

### Renal function studies

Plasma (pCr) and urinary creatinine (uCr) and proteinuria were determined with commercial kits based respectively on the Jaffe reaction and the Bradford method, following the manufacturer’s instructions (BioAssay System, Hayward, CA USA). Glomerular filtration rate was estimated by the creatinine clearance (ClCr), using the formula: ClCr = uCr × UF/pCr, were UF stands for urine flow.

The furosemide stress test (FST) was used to evaluate tubular function, as previously described^17^. Briefly, a single dose of furosemide (20 mg kg^−1^, i.p.) was administered to rats, and they were immediately allocated in metabolic cages to collect individual urine during the following hour. Urine volume and K^+^ excretion (LAQUAtwin B-731 Compact K^+^ meter, Horiba Scientific, Kyoto, Japan) were measured as indicators of tubular performance. Rats with damaged tubuli show lower furosemide-induced urinary volume and K^+^ excretion, compared to control rats^17^.

### Histological studies

Paraformaldehyde-fixed kidney samples were immersed in paraffin, cut into 5 μm-thick slices and stained with haematoxylin–eosin and with Periodic acid–Schiff. Whole-kidney images were obtained with photographs taken using the DotSlide virtual microscopy technique (Olympus BX51, Olympus Iberia, Barcelona, Spain). Images were analysed with the Olyvia Software (Olympus Iberia).

Tissue damage was quantified using a severity score, as previously described^38^. Briefly, 10 external medullar fields [i.e. the area damaged by cisplatin^37,39^] were chosen from dot slide images of each rat, and each field was divided into 6 sections. In each section, four parameters were evaluated: necrosis, tubular dilatation or atrophy, hyaline deposits and epithelial disorganization. A score of 0–3 was assigned to each parameter in a blind manner, according to the following criteria: 0, normal histology; 1, alteration in up to one-third of the section; 2, alteration from one-third to two-thirds of the section; 3, alteration in the whole area of the section. Section scores were added to give a field score (maximal score per section = 12). The average score of 10 fields was used for each kidney specimen.

### Measurement of urinary biomarkers

ELISA kits were used to measure urinary levels of KIM-1 (Cusabio, Wuhan, China), NGAL (Bioporto, Hellerup, Denmark), albumin (Abcam), transferrin (Abcam, Cambridge, UK), TIMP-2 (Abcam) and IGFBP-7 (Cloud Clone, Houston TX, USA), following the manufacturer instructions. The product of [TIMP-2]*[IGFBP7] urinary concentrations is marketed as NephroCheck®, and improves the diagnostic accuracy and outcomes of AKI patients. We have chosen these biomarkers due to previous studies by our research group^21^ and others^36,40^ which show their increased urinary levels in AKI patients and in different experimental models of AKI.

### Statistics and reproducibility

Statistical analysis was performed using the GraphPad Prism 7 software (San Diego, CA USA). Data normal distribution was evaluated with the Shapiro–Wilk normality test. Data are shown as mean ± standard error of the mean and dot blots. One-way ANOVA with Bonferroni’s test (for data with normal distribution) or Dunn’s test (for data with non-normal distribution) were performed to compare experimental groups; ROC curves were generated to analyse the ability of biomarkers (i.e., R0, R1 and R2) to predict predisposition to renal damage (i.e., development of a new AKI).Their areas under the curve (AUC) were calculated and compared with that of a hypothetical marker with zero diagnostic capacity (AUC = 0.50). Spearman correlations of biomarker level at predisposition stage with the severity of the subsequent AKI (i.e., pCr and ClCr values) were also calculated. *P* < 0.05 was considered statistically significant. N = 6 for each group and time point.

## Supplementary Information

Supplementary Figure 1.

## Data Availability

The data that support the findings of this study are available from the corresponding author upon reasonable request.
