# Supplementary material for: Biomarkers of persistent renal vulnerability after acute kidney injury recovery
Source: Sci Rep. 2021 Oct 27;11:21183. doi: 10.1038/s41598-021-00710-y (PMC8551194; doi:10.1038/s41598-021-00710-y)
Supplement: Supplementary file 1 — Supplementary Figure 1. [file 41598_2021_710_MOESM1_ESM.docx]

**
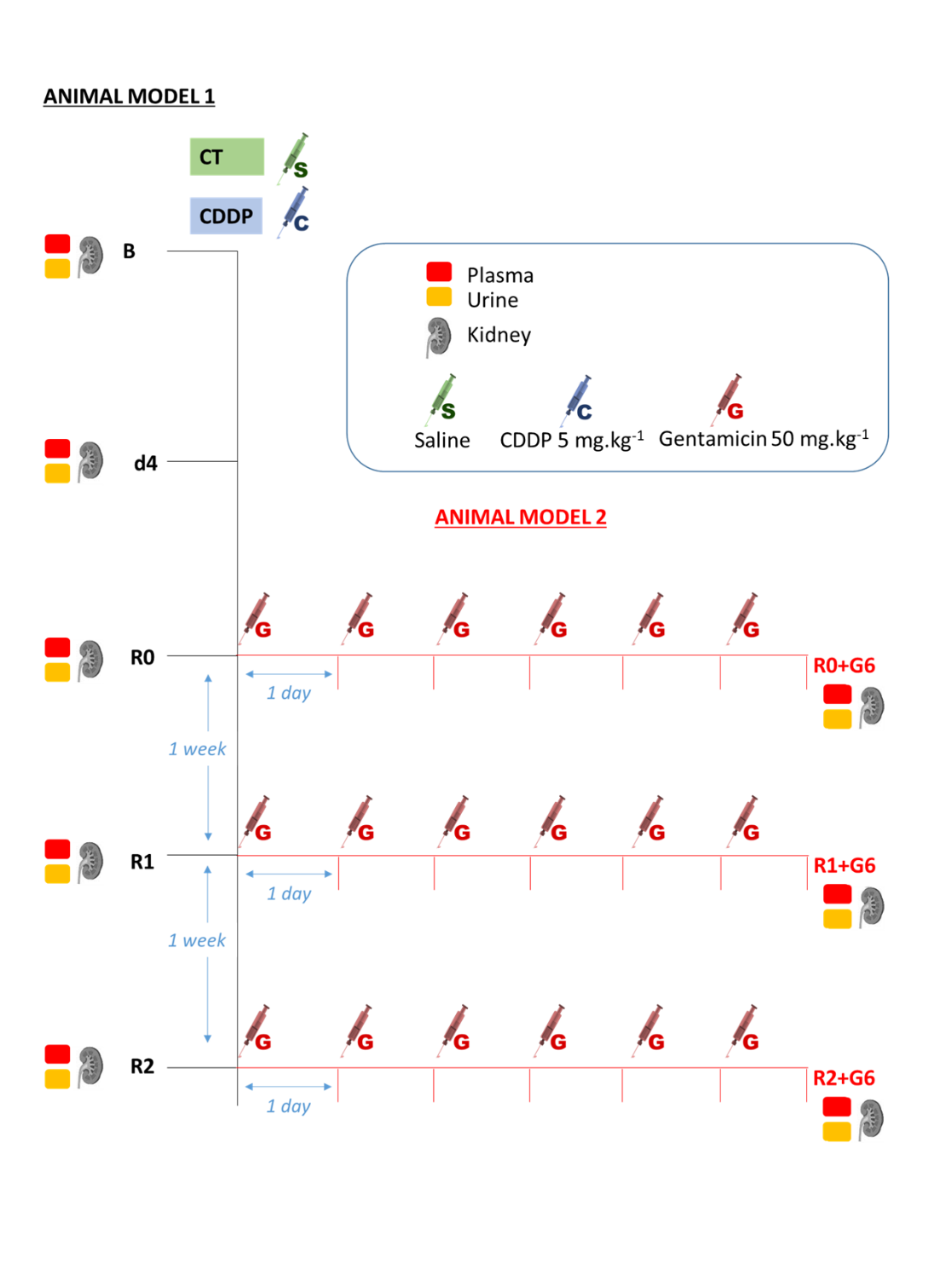
**

**Supplementary Figure 1. Temporal diagram of the experimental protocols and plasma, urine and kidney tissue sampling times**. B: basal; CDDP: cisplatin treatment; CT: control; d4: day of maximum kidney damage after cisplatin treatment; R0: day of recovery; R0+G6: six days of gentamicin treatment after R0; R1: one week after recovery; R1+G6: six days of gentamicin treatment after R1; R2: two weeks after recovery; R2+G6: six days of gentamicin treatment after R2
